# The Failure of Incipient Insanity Bills

**Published:** 1914-06-20

**Authors:** 


### The Hospital

The Workers' Newspaper of Administrative Medicine and Institutional Life, Administration, National Insurance and Health.

No. 1461, Vol. LVI. SATURDAY, JUNE 20, 1914. PRICE ONE PENNY.

### THE FAILURE OF INCIPIENT INSANITY BILLS.

There was a letter from Dr. Armstrong-Jones in the *Times* of June 15 calling attention to the fact that the Medico-Legal Society—or rather a special committee of that body—has just drafted a Bill "for the treatment of cases of early mental unsoundness without the stigmatising medical certificate and free from its legal disabilities." The object is a praiseworthy one, and if it could be attained would undoubtedly be of great value to those who, suffering from insanity in its early stages, can be brought back to their normal without the legal procedure which is at present necessitated. It is not possible, of course, to criticise the Bill in detail at present, as it has not apparently yet been made public; but we at once venture the criticism that whatever form it takes it is likely to defeat its own objects. For it is obviously to be a Bill which will take official and legal cognisance of incipient or "borderland" cases; and if cases are to come within this category it will follow that they will have to be in some way notified to the central authority—say, to the Board of Control. It may not be so stated in the Bill, but we may infer that this is likely to be one of the first results. Then the happy stage of notification will be added to the present certification, and the public will then be able to include a larger number of the mentally afflicted in its Index Expurgatorius of the certified or notified.

At the present time a quite considerable latitude is permitted to medical men as to the type of case which they may allow to remain in the category of the "non-certifiables." Anyone who has even a rudimentary acquaintance with lunacy practice is aware that hundreds, nay thousands, of cases are being treated in private and not in institutional care, uncertified. It is for the most part a question of means, and those who are able to pay for medical and nursing attention can obtain it without having recourse to the assistance of institutions for the mentally deranged.

Is it, then, the object of the Bill to free medical men and others from the restrictions of Section 315 of the Lunacy Act of 1890, which makes it a misdemeanour to "receive or detain a lunatic, or alleged lunatic," except under the provisions of the Act, that is if the person so doing receives payment for taking charge of the patient? But this section was obviously framed for the protection of the patient and to do away with the abuses which arose where there was no adequate supervision of the person of unsound mind by some such body as the Board of Control. Human nature has not greatly changed since that clause was framed, and it is still found to be useful in those too numerous instances where persons in charge of mental patients have lamentably failed to discharge their trust. It may be admitted that those who are desirous of doing away with the legal obligations which now exist are disinterested, but the fact remains that there are many others who would take advantage of any slackening of supervision. Yet it is to this section of the Act that Dr. Armstrong-Jones makes especial reference as entailing particular difficulties in the carrying out adequately of the treatment of incipient insanity.

Everyone would welcome some measure which would remove the disabilities of certification, and we hope that the Medico-Legal Society has found a way out of the difficulties which beset the path of legislators who deal with the problem of incipient insanity. The Commissioners in Lunacy—now the Board of Control—have had a vast experience of the working of the Lunacy Act, and we do not know that they have found it expedient to relax the stringency of Section 315 of that Act. In the circumstances it is obvious that anyone suggesting fresh legislation must needs walk warily and be careful that the remedy suggested is not worse than the disease.

The so-called "stigma" of insanity will not be removed by Incipient Insanity Bills. The feeling of doubt in regard to the efficiency of those who have once been insane is based upon practical experience of the unreliability of *some* of those who have been the subject of a mental breakdown, and this part is made to include the whole, as is so often the case in popular logic; just as "certification" would be made to include "notification." Then, again, there is the *odium theologicum*—acting perhaps, to adopt the phraseology of the Freudian school, in many cases as a repressed complex, but none the less influencing many people—the feeling that the insane are possessed by devils and that they are, therefore, not desirable company for their more sane—or less insane—brothers! As time goes on, public opinion may be educated to the stage of classing mental disorders with other physical troubles and of realising that in certain cases mental stability may be permanently recovered. Even then the public may feel some hesitation in regard to placing reliance upon those who have been for a time mentally unsound, realising what is apparently an accepted scientific doctrine—that where there has been a mental breakdown there is some nervous instability which renders the individual less able to bear the strain entailed by so many of the occupations in which members of a civilised community have to engage. In the light of these facts the text of the new Bill must be awaited.

